# Prevalence and Persistence of Antibiotic Resistance Determinants in the Gut of Travelers Returning to the United Kingdom is Associated with Colonization by Pathogenic Escherichia coli

**DOI:** 10.1128/spectrum.05185-22

**Published:** 2023-05-31

**Authors:** Timothy J. Dallman, Saskia Neuert, Cristina Fernandez Turienzo, Michelle Berin, Emily Richardson, Pablo Fuentes-Utrilla, Nicholas Loman, Saheer Gharbia, Claire Jenkins, Ron H. Behrens, Gauri Godbole, Michael Brown

**Affiliations:** a Gastrointestinal Bacteria Reference Unit, UK Health Security Agency London, United Kingdom; b Faculty of Veterinary Medicine, Institute for Risk Assessment Sciences (IRAS), Utrecht University, Utrecht, the Netherlands; c Gut Microbes and Health, Quadram Institute Bioscience, Norwich, United Kingdom; d National Institute for Health Research Health Protection Research Unit in Gastrointestinal Infections, University of Liverpool, Liverpool, United Kingdom; e Hospital for Tropical Diseases, University College London Hospitals NHS Foundation Trust, London, United Kingdom; f Division of Infection and Immunity, University College London, London, United Kingdom; g Faculty of Life Sciences and Medicine, King’s College London, London, United Kingdom; h Institute of Microbiology and Infection, University of Birmingham, Birmingham, United Kingdom; i MicrobesNG, Birmingham, United Kingdom; j National Institute for Health Research Health Protection Research Unit in Genomics and Enabling Data, Warwick University, United Kingdom; k Department of Clinical Research, Faculty of Infectious and Tropical Diseases, London School of Hygiene and Tropical Medicine, London, United Kingdom; University at Albany

**Keywords:** *Escherichia coli*, antibiotic resistance, diarrhea

## Abstract

The gut microbiota constitutes an ideal environment for the selection, exchange, and carriage of antibiotic resistance determinants (ARDs), and international travel has been identified as a risk factor for acquisition of resistant organisms. Here, we present a longitudinal metagenomic analysis of the gut resistome in travellers to “high-risk” countries (Gutback). Fifty volunteers, recruited at a travel clinic in London, United Kingdom, provided stool samples before (pre-travel), immediately after (post-travel), and 6 months after their return (follow-up) from a high-risk destination. Fecal DNA was extracted, metagenomic sequencing performed and the resistome profiled. An increase in abundance and diversity of resistome was observed after travel. Significant increases in abundance were seen in antimicrobial genes conferring resistance to macrolides, third-generation cephalosporins, aminoglycosides, and sulfonamides. There was a significant association with increased resistome abundance if the participant experienced diarrhea during travel or took antibiotics, but these two variables were co-correlated. The resistome abundance returned to pre-travel levels by the 6-month sample point but there was evidence of persistence of several ARDs. The post-travel samples had an increase in abundance Escherichia coli which was positively associated with many acquired resistant determinants. Virulence and phylogenetic profiling revealed pathogenic E. coli significantly contributed to this increase abundance. In summary, in this study, foreign travel remains a significant risk factor for acquisition of microbes conferring resistance to multiple classes of antibiotics, often associated with symptomatic exposure to diarrhoeagenic E. coli.

**IMPORTANCE** A future where antimicrobial therapy is severely compromised by the increase in resistant organisms is of grave concern. Given the variability in prevalence and diversity of antimicrobial resistance determinants in different geographical settings, international travel is a known risk factor for acquisition of resistant organisms into the gut microbiota. In this study, we show the utility of metagenomic approaches to quantify the levels of acquisition and carriage of resistance determinants after travel to a “high-risk” setting. Significant modulation to the resistome was seen after travel that is largely resolved within 6 months, although evidence of persistence of several ARDs was observed. Risk factors for acquisition included experiencing a diarrheal episode and the use of antibiotics. Colonization by pathogenic Escherichia coli was correlated with an increase in acquisition of antimicrobial resistance determinants, and as such established public health guidance to travelers on food and water safety remain an important message to reduce the spread of antibiotic resistance.

## INTRODUCTION

Rising antimicrobial resistance (AMR) in hospital and community settings is associated with grave health and economic consequences and continues to be the focus of reflection and sustained attention among clinicians. The presence of antibiotic resistance determinants (ARDs) in nature predates the use of antimicrobial therapy, but increased use of antibiotics and global travel are both well-recognized risk factors for acquisition of resistance (1–4). The prevalence and diversity of antimicrobial resistance genes varies in different geographical settings with many countries in the global south having higher rates of antimicrobial resistance driven by challenges in the adoption and enforcement of antimicrobial usage policies (5, 6). As such, international travel represents an important mode of dissemination of antimicrobial resistance determinants and have featured heavily in the origins of well-characterized recent clonal expansions of virulent Gram-negative bacteria (7–11).

Prospective studies among travelers have sought to quantify the risk of acquiring ARD-carrying bacteria associated with travel to high-risk countries, study the duration of colonization by resistant organisms, and measure the effect of antibiotic use on both (12–19). Most studies have reported that ARD persistence beyond 3 months of travel is unusual (12). However, these studies have predominantly employed culture-based methods, or used predefined primers targeting common resistance-conferring alleles. The inherent bias in selective culture and the use of targeted panels may lead to an underestimate of the true burden of acquisition and persistence (17).

There is limited understanding about the effect of travel on the composition of the gut microbiota in humans and its potential correlation with ARD acquisition and persistence. One recent longitudinal study (20), which used 16S rRNA gene amplicon sequencing to quantify the gut microbial diversity in travelers, showed that the composition of their gut microbiota does not affect their risk of acquiring cephalosporin-resistant Enterobacteriaceae when travelling to a high-risk country like India. Metagenomic surveys of fecal and other host-associated microbiomes have been used to study host-microbial interactions in health and disease (21). These techniques obviate some of the limitations of culture-based methods and allow a more thorough exploration of the fecal microbiome. Specifically, the technology can be used to explore the resistome by querying established databases of resistance-associated sequences (22, 23). Studies using metagenomics sequencing, performed on environmental samples like water and toilet waste, have shown high abundance and diversity of genes encoding antimicrobial resistance in South Asia (24, 25). Similarly, a recent study among Dutch travelers suggested the destination of travel is a strong predictor of the type of antimicrobial gene families acquired and the magnitude of resistance acquisition (26).

Early presumptive self-treatment for travelers’ diarrhea (TD) has been shown to be effective in reducing the duration of acute travelers’ diarrhea, and is used as a standby therapy for selected individuals attending travel clinics (12, 27–30). However, there is also evidence suggesting that the use of antibiotics and episodes of diarrhea may increase the risk of carrying extended-spectrum β-lactamase (ESBL) producers and other antibiotic-resistant bacteria, thus posing a threat to the individual’s health and favoring the further spread of AMR (13, 18, 19, 31, 32). The primary objective of this study was to use metagenomics sequencing to detect the presence of and quantify known ARDs in the fecal microbiota before and after travel to a high-risk country. The secondary objective was to explore associations between antimicrobial treatments, travel-acquired illness, and acquisition and persistence of resistance elements.

## RESULTS

**FIG 1:**
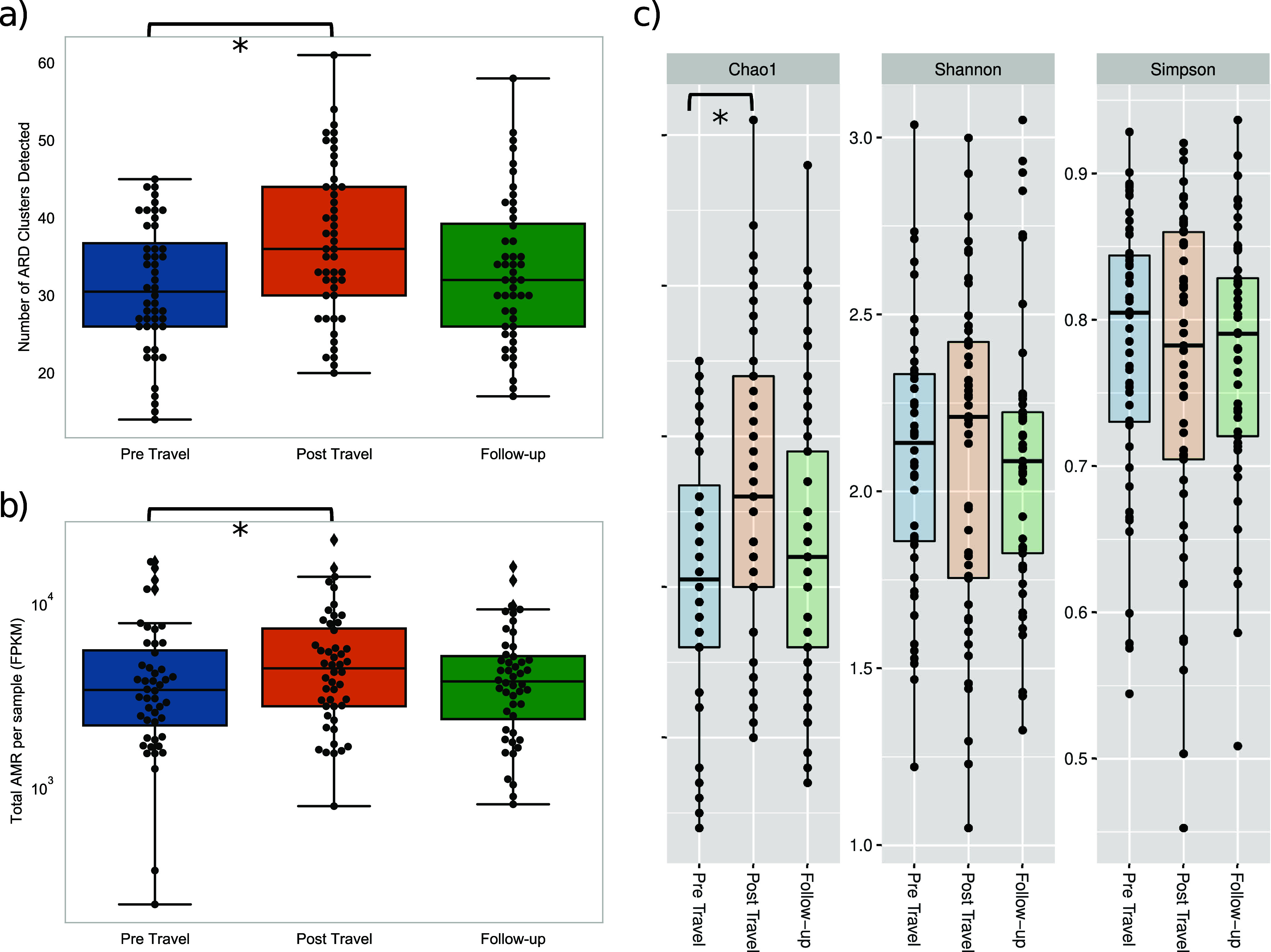
Number of ARDs (a) and abundance (b) of resistome detected in pre-travel, post-travel, and follow-up samples. (c) Alpha diversity per sample type based on Chao1, Shannon, and Simpson’s metrics.

The impact of travel on resistome diversity was assessed by comparing the number of individual ARDs detected at the three time points. A median of 30.5 individual ARDs (IQR = 10.75) were detected in the pre-travel specimens compared to 36 ARDs (IQR = 14) in the post-travel, and 32 ARDs (IQR = 13.25) in the follow-up samples. There was a wide range in the number of ARDs detected within each category (pre-travel:14 to 45 post-travel:20 to 61, follow-up:17 to 58). The increase in ARDs detected post travel was significant (Wilcoxon rank-sum test, *P* < 0.05) (Fig. 1a). Normalized resistome abundance was further expressed as fragments per kilobase reference per million bacterial fragments (FPKM). Resistome abundance was significantly higher in post-travel samples compared to pre-travel samples (Wilcoxon rank-sum test, *P* < 0.05) (Fig. 1b). Within specimen type, resistome diversity (alpha diversity) was calculated based on Chao1, Shannon, and Simpson’s metrics with significant differences observed between pre-travel and post-travel with the Chao1 metric (Wilcoxon rank-sum test, *P* < 0.05) but all metrics exhibiting a higher median alpha diversity measure in post-travel samples (Fig. 1c).

**FIG 2:**
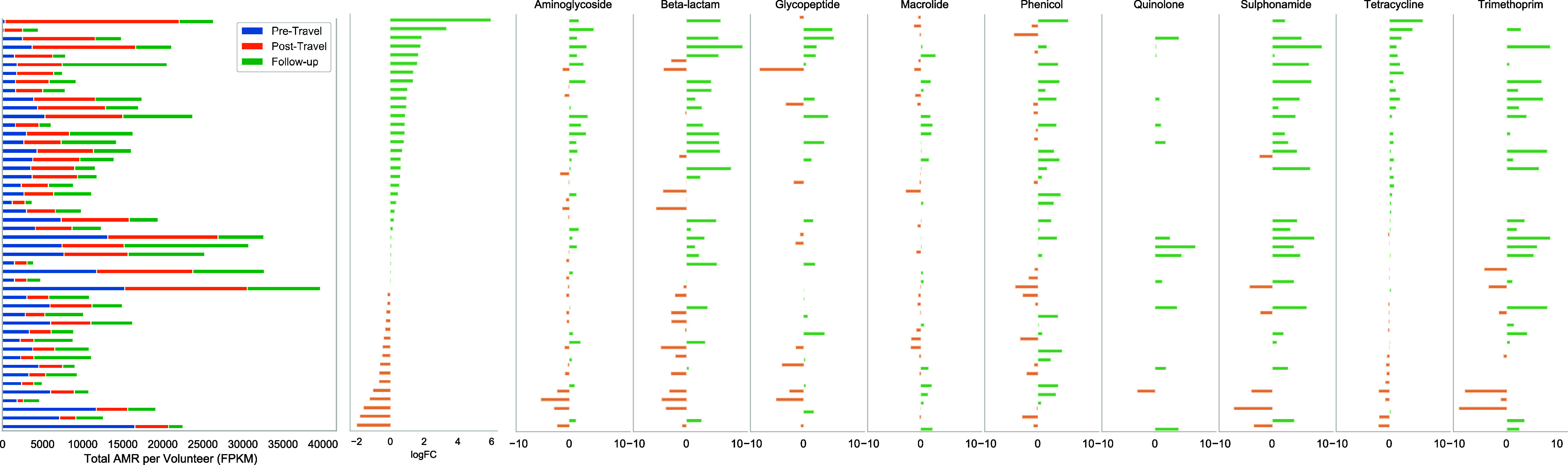
Left panel: total AMR per volunteer per time point expressed by fragments per kilobase reference per million bacterial fragments (FPKM). For each volunteer the log_2-fold_ change between pre-Travel and post-Travel samples was calculated based on total abundance (central panel) and delineated by antimicrobial class (right panel).

Thirty-one participants (64.6%) had an increase in resistome abundance immediately after travel compared to their pre-travel sample (Fig. 2, left panel). For 21 out of these 31 (67.7%), the abundance remained above that of the pre-travel specimen in the follow-up samples. With respect to changes in abundance by class, 26 (54.2%) participants had an increase in abundance of Aminoglycosides after travel, 24 (50.0%) participants had an increase in abundance of Beta-lactams, 19 (39.6%) participants an increase in Glycopeptide abundance, 23 (47.8%) participants had an increase in Macrolide abundance, 26 (54.2%) an increase in Phenicol abundance, 14 participants (29.2%) had an increase in Quinolone abundance, 24 (50.0%) participants had an increase in Sulphonamide abundance, 28 (58.3%) participants had an increase in Tetracycline abundance, and 24 (50.0%) had an increase in Trimethoprim abundance in their post-travel specimen (Fig. 2, right panel).

Tetracycline ARDs were ubiquitously present and predominated the resistome of most participants with a median proportion of 70.8% in pre-travel samples (IQR = 27.4%), 78.6% in post-travel samples (IQR = 23.3%) and 78.7% in follow-up samples (IQR = 19.8%). The next most abundant ARDs conferred resistance to macrolides with a median proportion of 13.7% in pre-travel samples (IQR = 16.3%), 9.6% in post-travel samples (IQR = 9.5%), and 10.1% in follow-up samples (IQR = 12.5%). The median proportion of ARDs conferring resistance to the other classes (Aminoglycosides, Beta-lactams, Colistin, Fosfomycins, Fusidic Acid, Glycopeptides, Nitroimidazole, Phenicols, Pseudomonic Acid, Quinolones, Sulphonamides, and Trimethoprim) were less than 5% in pre-, post-travel, and follow-up samples (Fig. S1).

### Abundance, acquisition, and carriage of antimicrobial resistance determinants.

**FIG 3:**
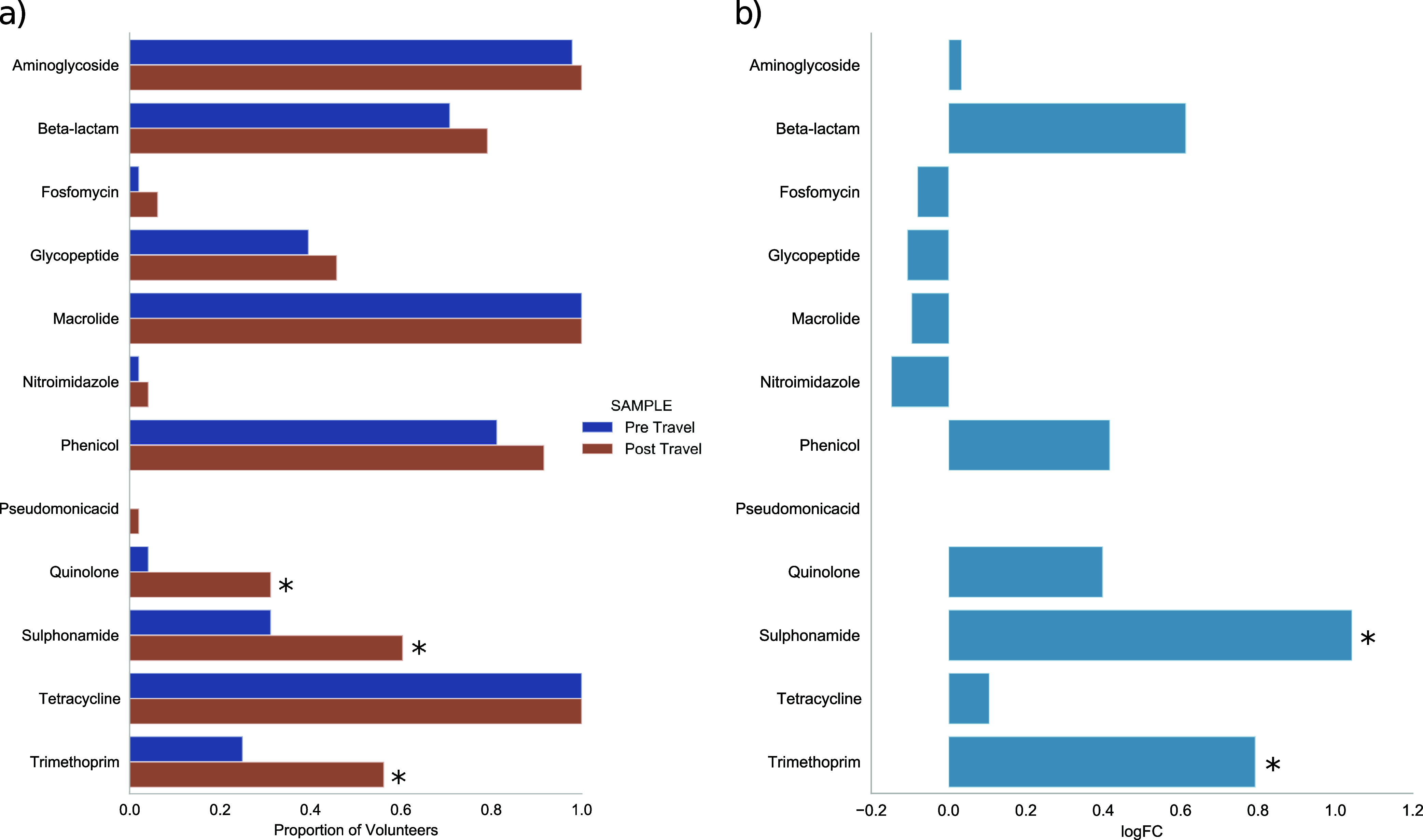
(a) Proportion of volunteers with ARDs detected stratified to antimicrobial class in pre- and post-travel samples and (b) differential abundance of ARDs by antimicrobial class in pre- and post-travel samples.

In the pre-travel sample set, ARDs conferring resistance to 11/14 of the assayed antimicrobial classes were seen in at least one participant with proportions ranging from 0% (colistin, fusidic acid, mupirocin) to 100% (macrolides and tetracyclines) of volunteers. In addition, four other antimicrobial classes were present in the pre-travel set at a proportion of greater than 50%, aminoglycosides (97.9%), phenicol (81.2%), macrolides (98%), and beta-lactams (70.8%). When comparing participants pre- to post-travel samples, for the 12 antimicrobial classes detected in the post-travel samples all were of equal or increased proportion in the post-travel sample set with sulfonamides, quinolones and trimethoprim classes all reaching a significant difference (McNemar test, *P*-value < 0.05) (Fig. 3a). To explore the changes in resistome abundance in pre-travel and post-travel samples differential abundance analysis was performed with a significant increase in abundance of sulfonamides (+1.04 logFC) and trimethoprim (+0.79 logFC) in the post-travel sample set (*P* value <0.05) (Fig. 3b).

A total of 181 antimicrobial gene clusters were identified in the pre- and/or post-travel samples. Of these, 114 showed in an increase in prevalence in the post-travel samples compared to the pre-travel with 21 showing no difference and 46 higher in the pre-travel samples. In total, 33/48 (68.8%) of participants had an increase in detected antimicrobial gene clusters in their post-travel sample compared to the pre-travel sample with a median increase of 9 ARDs. In addition, 72 antimicrobial gene clusters were present in both the post-travel and follow-up samples but absent in the pre-travel sample for at least one participant. The median number of acquired antimicrobial gene clusters that persisted into the follow-up sample was 3 (IQR = 2.5) per participant. 11 antimicrobial gene clusters exhibited potential carriage in five or more volunteers, the macrolides *mdf(*A) (*n* = 13, 27.1%), *erm*(G) (*n* = 6, 12.5%), and *mef(A)* (*n* = 5, 10.4%), the beta-lactams *bla*_TEM-1A_ (*n* = 7, 14.6%) and *cfxA* (*n* = 5, 10.4%), the tetracyclines *tet(Q)* (*n* = 6, 12.5%), *tet(A*) (*n* = 5, 10.4%) and *tet(X)* (*n* = 5, 10.4%), the aminoglycosides *aph(*6*)-ld* (*n* = 8, 16.7%) and *aph(3′)-IIa* (*n* = 7, 14.6%), and the phenicol *catS* (*n* = 6, 12.5%).

**FIG 4:**
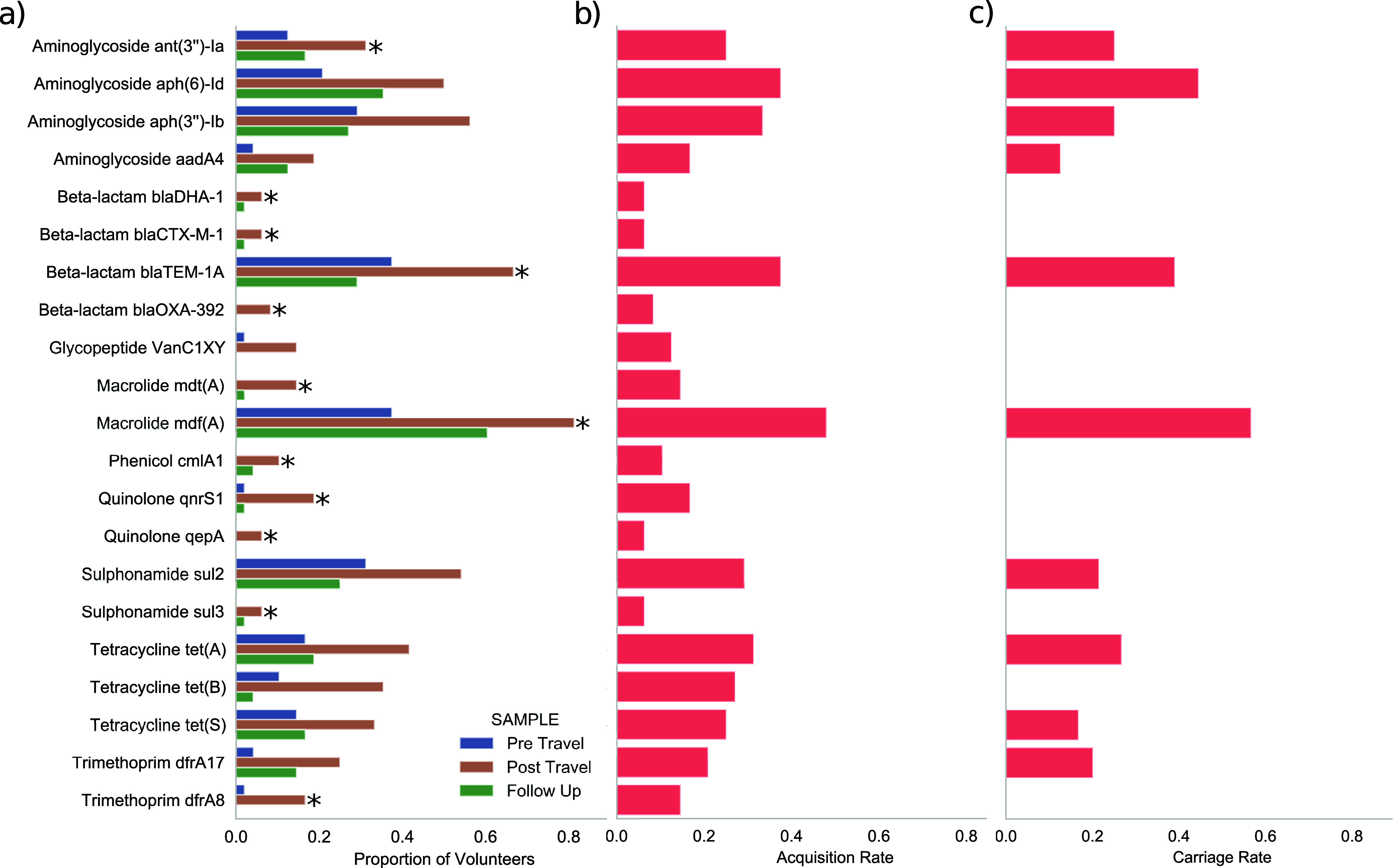
(a) Proportion of ARDs with significant differences in proportion and/or abundance between in pre- and post-travel samples stratified to antimicrobial gene. The star label represents those ARDs that were significantly differential abundant (including structural zeros). (b) Acquisition rate (proportion positive in post-travel samples that were negative in the corresponding pre-travel sample) for these ARDs and (c) carriage rate (proportion still positive in follow-up samples that were negative in the corresponding pre-travel sample) of these ARDs.

There were 15 antimicrobial gene clusters that were significantly more likely to be present in a participant’s post-travel sample compared to their pre-travel sample (McNemar test *P*-value < 0.05). These included the aminoglycosides *aph(6)-Id*, *ant(3′)-Ia*, *aph(3′')-Ib*, and *aadA4*, the beta-lactam *bla*_TEM-1A_, the glycopeptide *VanC1XY*, the macrolides *mdf(A)* and *mdt(A)*, the quinolones *qnrS1*, the sulfonamide *sul2*, the tetracyclines *tet(A)*, *tet(B)*, and *tet(S)*, and the trimethoprim resistance genes *dfrA8* and *drA17* (Fig. 4a). When comparing differential abundance of antimicrobial gene clusters in the pre- and post-travel sets *aph(6)-Id* (log FC +1.1), *bla*_TEM-1A_ (log FC +1.4), *mdf(A)* (log FC +1.3), *mdt(A)* (log FC +0.13) were also in significantly higher abundance in the post-travel samples in addition to the beta-lactams *bla*_DHA-1_ (log FC +0.05), *bla*_CTX-M-1_ (log FC +0.03) and *bla*_OXA-392_ (log FC +0.4), the phenicol gene cluster *cmlA1* (log FC +0.11), the sulfonamide *sul3* (log FC +0.08) and the quinolone resistance gene cluster *qepA* (log FC +0.07) (Fig. 4a).

Acquisition rates for these resistance gene clusters of increased prevalence or abundance (proportion positive in post-travel samples that were negative in the corresponding pre-travel sample) ranged from 0.48 (mdf(A)) to 0.063 (*bla*_DHA-1_, *bla*_CTX-M-1_, *qepA* and *sul3*) with a median rate of acquisition of 0.167 (Fig. 4b). Of the resistance gene clusters of increased prevalence or abundance in the post-travel samples, 11/21 were not present in the corresponding follow-up sample. Of the 10 that were still detectable in participants’ follow-up samples, carriage rates (proportion still positive in follow-up samples that were negative in the corresponding pre-travel sample) ranged from 0.125 for *aadA4* to 0.56 for *mdf(A)* (Fig. 4c).

### Comparison of pre-travel, post-travel, and follow-up resistome composition and associated risk-factors.

To assess the variation in resistome composition nonmetric multidimensional scaling (NDMS) ordination was performed on normalized ARD cluster read-counts using the exploratory variables, sample type (post/pre/follow-up) and whether the sample was from the same participant (Fig. S2). The resistomes from samples from the same participant and samples from the same time points were both significantly clustered with an effect size of 60.3% (ADONIS, *P*-value 0.001) and 2.2% (ADONIS, *P*-value 0.016), respectively.

Variation in the post-travel resistome was analyzed to explore any potential trends with region of travel, the use of an antimalarial prophylactic, the use of antibiotics during travel and whether the participant had TD. Samples from participants that experienced TD during their travels were significantly clustered (ADONIS, *P*-value 0.017, effect size 4.3%) with the other variables showing no significant clustering in resistome composition. No significant difference was detected in alpha diversity associated with any of these exploratory variables in the post-travel samples.

With respect to traveler’s diarrhea 23/48 participants experienced an episode during travelling. Comparing resistome abundance, those who experienced TD had a significant increase in abundance (Mann-Whitney U-test, *P*-values < 0.05) of ARDs in their stool sample. ARDs with significant differential abundance between participants that had an episode of TD and those that had not were sought, with the use of an antimalarial prophylactic, the use of antibiotics, and continent of travel included as covariates. A single antimicrobial gene cluster was significantly enriched in the participants who experienced TD, the macrolide *mef(A)* (logFC +1.50).

Ten out of 48 individuals consumed antibiotics during their initial travel period; eight were prescribed ciprofloxacin and two azithromycin and there was a significant increase in resistome abundance in the group that consumed antibiotics (Mann-Whitney U-test, *P*-values < 0.05). Antimicrobial gene clusters with significant differential abundance between participants that had consumed antibiotics and those that had not were sought with the use of an antimalarial prophylactic, whether the participant had TD during travel and continent of travel included as covariates. A single antimicrobial gene cluster was significantly enriched in the participants who consumed antibiotics, the macrolide *lsa(C)* (logFC +1.62).

Twenty-one out of 48 individuals consumed an antimalarial prophylactic before their initial travel period; 18 were prescribed atovaquone/proguanil and chloroquine; doxycycline and mefloquine were prescribed to three individual patients. There was no significant difference in resistome abundance between those who had consumed an antimalarial prophylactic and those who had not (Mann-Whitney U-test, *P*-values > 0.05) and no antimicrobial gene cluster that significantly differed in abundance.

With respect to destination of travel, 19/48 traveled to Africa, 14/48 traveled to the Americas and 15/48 traveled to Asia. Comparing resistome abundance, there was no significant difference attributable to travel destination (Kruskal–Wallis, *P*-values > 0.05). There were also no antimicrobial gene clusters deemed significantly differentially abundant between travel destinations.

### Travel and the effect on the microbiome.

Within specimen type, microbiome diversity (alpha diversity) was calculated with significant differences observed between pre-travel and follow-up with the Shannon metric (Wilcoxon rank-sum test, *P* < 0.05) (Fig. S3a). To assess the variation in microbiome composition NDMS, ordination was performed using the exploratory variables, sample type (post/pre/follow-up), and whether the sample was from the same participant. The microbiomes from samples from the same participant and samples from the same time points were both significantly clustered with an effect size of 60.6% (ADONIS, *P*-value < 0.001) and 2.8% (ADONIS, *P*-value 0.002), respectively (Fig. S3b). A single microbial species, Escherichia coli (logFC +4.87), was significantly in increased abundance between pre- and post-travel samples which was reflected in 30/48 (62.5%) volunteers exhibiting an increase in E. coli abundance post-travel (Fig. S4). Markers indicative of E. coli pathogenicity were profiled in each sample. In the pre-travel samples specimens, 1/48 was positive for pathogenic E. coli, an Enteropathogenic E. coli (EPEC). In the post-travel samples, 17/48 specimens were positive for pathogenic E. coli, 13 with markers for EPEC, eight with markers for Enteroaggregative E. coli (EAEC), two with markers for Enteroinvasive E. coli (EIEC), and one specimen with markers for Enterotoxigenic E. coli (ETEC). Six out of 17 specimens were positive for markers indicative of multiple pathotypes, suggesting infection with multiple strains of E. coli or strains with a hybrid pathotype profile, with four positive for EPEC in conjunction with EAEC, one positive for EPEC in conjunction EIEC, and one positive for EPEC in conjunction with both EAEC and ETEC. In the follow-up samples, 7/48 specimens were positive for markers of pathogenetic E. coli (2 EPEC, 3 EAEC, 1 ETEC, and 1 EPEC/STEC). Of the 17 participants colonized with pathogenic E. coli in their post-travel specimen, nine reported travelers’ diarrhea while eight did not, with EIEC and ETEC only found in participants who reported TD. Those reporting gastrointestinal illness had a significantly higher abundance of E. coli then those did not (Kruskal-Wallis, *P*-value < 0.05).

**FIG 5:**
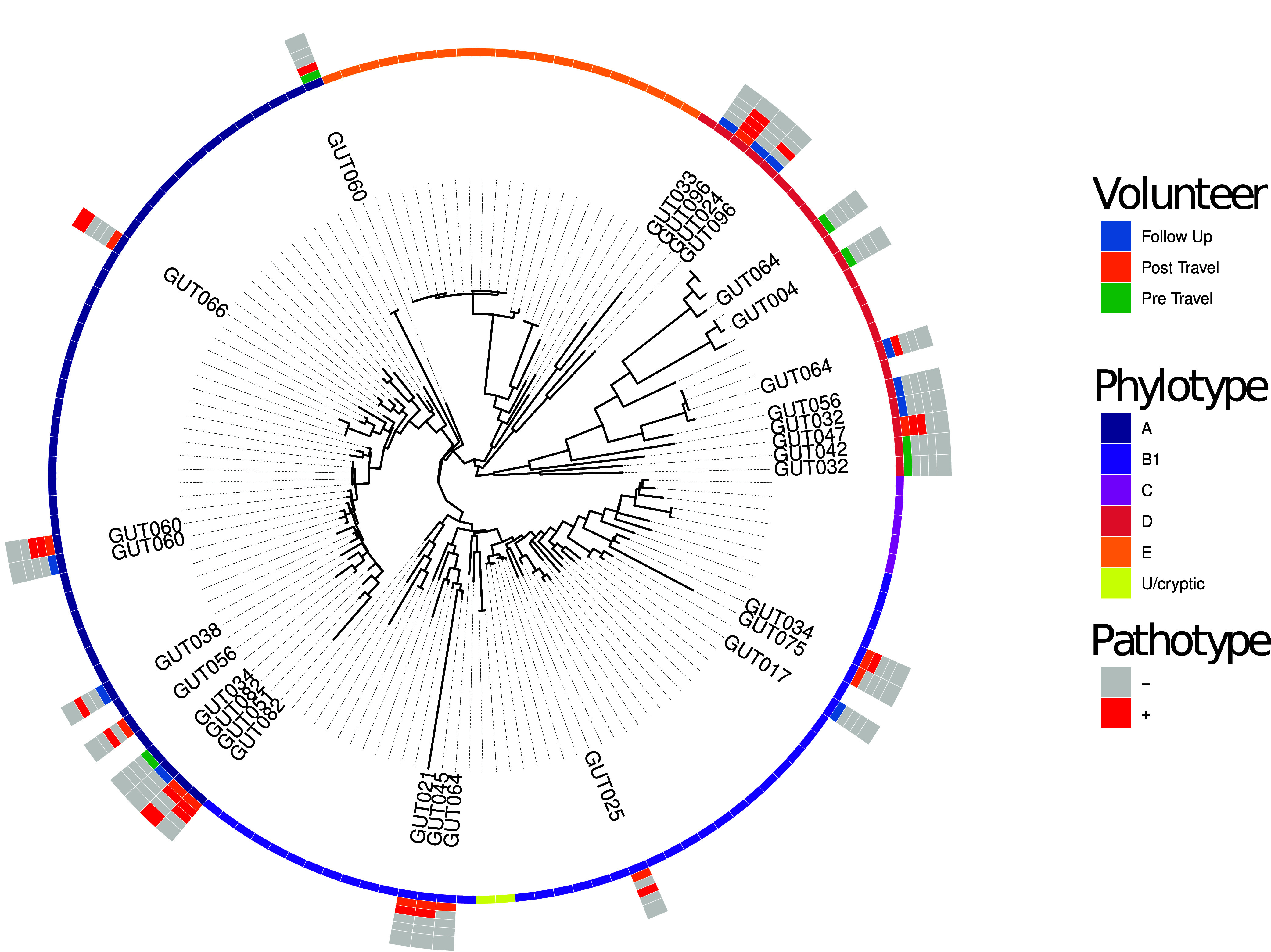
Phylogenetic analysis of 29 samples where there was adequate E. coli abundance for strain profiling compared to 105 isolates representing the Clermont E. coli phylotypes (A to G and U. Taxa are labeled by participant. From inner to outer the concentric rings are colored by phylotype, specimen type, EPEC status, EAEC status, ETEC status, and EIEC status.

Twenty-nine samples (6 pre-travel, 13 post-travel, 10 follow-up) from 20 participants had an adequate abundance of E. coli assigned reads for phylogenetic strain profiling (Fig. 5). Strains were identified across several phylotypes with most strains clustering in phylotypes A and B1. Where E. coli was identified longitudinally across at least two of the pre-travel, post-travel, and follow-up, the strains were generally phylogenetically distinct suggesting E. coli colonization in the gut can be transient. One exception was the E. coli identified in the post-travel and follow-up specimens of volunteer (Gut60) which phylogenetically clustered; however, the post-travel specimen had markers of EPEC and EAEC while the follow-up sample had no pathotype markers (Fig. 5).

To identify which taxa were associated with individual resistance determinants, differentially abundant taxa associated with each ARD that was of significantly increased abundance and/or proportion in the post-travel data set were sought. Testing those antimicrobial gene clusters that were present in at least five participants (*n* = 16), 12 were significantly associated with an increased abundance in the E. coli (*cmlA1*, *dfrA8*, *dfrA17*, *qnrS1*, *tet(A)*, *tet(B)*, *aadA4*, *ant(3′')-Ia*, *aph(6)-Idl*, *aph(3′)-Ib*, *sul2*, *bla_TEM-1A_*, *mdf(A)*) and three were associated with a significant increase in Enterococcus faecalis abundance (*tet(S)*, *VanC1XY* and *mdt(A)*)

## DISCUSSION

The prevalence of microorganisms within people and animals harboring genes conferring resistance to antimicrobials differs in different regions of the world (33). Drivers for this discrepancy are in part due to differences in antimicrobial stewardship, differences in transmission dynamics within and between hosts, and within low and middle income countries, and often the lack of sanitation and inadequate infection prevention to control the spread of microbes (6, 34). It has been previously demonstrated that international travel to regions with high antimicrobial resistance burden has been associated with the acquisition of bacteria that encode for resistance genes (e.g., ESBL-producing E. coli) but their persistence over time remains underexplored (26, 35).

This study utilized shotgun metagenomics to assess and quantify the risk of antimicrobial acquisition and carriage resulting from travel to regions of the world with a high burden of antimicrobial resistance (5). There was a significant increase in the number and abundance of antimicrobial resistance genes and resistome diversity in the post-travel samples, with levels returning to the pre-travel baseline in the 6-month follow-up sample set, suggesting that the enrichment of the resistome following travel is largely temporary.

All antimicrobial classes that were not ubiquitous among participants increased in proportion in the post-travel set, and sulfonamides, quinolones, and trimethoprim resistance genes were significantly more likely to be present in a participant’s post-travel sample compared to their pre-travel sample. Twenty-one antimicrobial gene clusters increased in abundance to significant levels in the post-travel set compared to the pre-travel set and included nine different antimicrobial classes with several genes (e.g., qnrS, *bla*_CTX-M-1_) also reported as commonly acquired ARDS in a recent study of the resistome changes in Dutch travellers (26).

Based on known mechanisms of transfer and genomic context, the acquired ARDs could be broadly separated into those AMR elements that are intrinsic to certain species (e.g., *mdf(A)* chromosomally encoded in E. coli), those that are predominantly plasmid mediated (e.g., *qnrS1*), and those encoded on transposases that can be located on both chromosomes and plasmids (e.g., *bla*_TEM_). The majority of gene clusters were acquired at relatively low frequency in the post-travel population and acquisition and carriage of elements that were predominantly plasmid encoded was particularly low.

Individuals’ microbiomes across the three time points were significantly more similar to each other than between participants with the individual effect explaining over 60% of the variability in the samples. This trend was also seen in the resistome; although post-travel resistomes were more similar to each other than pre-travel or follow-up, the effect (2%) was much smaller than the effect of the individual. This suggests that the majority of the resistome, like the microbiome, is inherently stable and explained by determinants that are intrinsic to that core microbiome.

The only significant change in microbial abundance in the pre- to post-travel samples was a large increase in the E. coli in the post-travel samples, and this was shown to co-correlate with the majority of acquired resistance genes with the exception of *tet(S)*, *VanC1XY*, and *mdt(A)* which were both associated with a significant increase in *Enterococcus* abundance. When profiling the individual E. coli associated with each sample, we observed a propensity for colonization with pathogenic E. coli in the post travel sample. These were phylogenetically diverse and distinct to any E. coli present in the volunteers’ pre-travel or follow-up samples. This provides evidence that clearance of these pathogenic E. coli occurs, and their persistence is not contributing directly to the carriage of any antimicrobial determinants. Genes indicative of multiple pathotypes of E. coli were often detected in the same sample likely representing multiple strains of E. coli that had been ingested. It has previously been documented that those travelers who experience an episode of diarrhea were at greater risk of colonization with antibiotic resistance encoding bacteria (35) and a significant increase in resistome abundance was associated with TD in this study. Furthermore, the pathogenic E. coli identified in post-travel samples were detected in volunteers who more often reported TD.

Recent studies have suggested that the destination of travel significantly shapes the resistome (19, 26) but no effect on travel destination and resistome abundance or composition was observed in this study. This negative result can probably be explained by the high diversity in both travel destinations and ARDs detected, and the relatively small sample sizes thus limiting the statistical power to detect significant resistome changes. In addition, an effect of prophylactic use of antimalarials on the resistome was not observed.

This study aimed to be proof of principle of a longitudinal resistome study in travelers to high-burden AMR regions. While our criteria are broadly in-line with those reported elsewhere, we acknowledge that all regions display an overall heterogeneity in resistance burden. (5, 33). Future studies that allow more granular geographic comparisons of resistomes from all risk spectrums may help to understand the drivers of acquisition and persistence further.

The study design aimed to understand the risk of prolonged carriage of antimicrobial elements (at 6 months) that was hampered by several confounding effects, including many participants who traveled again in the period between their post-travel sample and their 6-month travel sample. Although overall carriage level was low, particularly in the antimicrobial gene clusters that were differentially present/abundant in the groups, at an individual level, participants on average showed persistence of three acquired genes into their follow-up sample. Given the above, it is difficult to untangle whether these represent carriage, independent acquisition, or absence of detection in the pre-travel sample. We also note that a significant proportion of participants (30%) reported suffering from a chronic condition or were pregnant which may have influenced the overall resilience of the microbiome and as such the resistome. The effect of such confounders on resistome acquisition and carriage should be studied in the future.

The evolution of antimicrobial resistance to different antibiotics has been realized in multiple ways in nature—hence, the vast number of genes (and alleles) that are available to microbes. As a result of this extensive repertoire of mechanisms available, their prominence in different settings or geographical regions also varies considerably (33). Conversely, at the antimicrobial class level the resistance to at least one antibiotic in that class is often at high levels and sometimes ubiquitous(26). Both these phenomena provide analytical challenges to assessing changes to the resistomes in broad settings such as international travel. In this study, antimicrobial genes were clustered at two broad levels of classification, antimicrobial class and antimicrobial gene cluster (90% sequence identity), to try and provide two perspectives, but further stratifications may be useful to capture more relevant antimicrobial pressures. While metagenomics provides a uniquely broad perspective on the resistome, the linkage between the resistance genes and the organism encoding it remains elusive with short-read sequencing, similarly detection of causative point mutations in mixed populations remains a challenge.

## MATERIALS AND METHODS

### Study design.

Participants were recruited for the study when they attended the pre-travel outpatient clinic at a large teaching hospital in London, United Kingdom. Consecutive travelers, with or without underlying conditions, attending the clinic for pre-travel advice and medication were invited to participate. Additionally, healthy individuals (such as research staff and students) responding to advertising and promotional events online and at the local universities’ campuses were recruited. Recruitment was completed between December 2015 and July 2016. A participant information leaflet (PIL) incorporating an invitation to take part in the study was provided to potential study subjects in person or by post. Individuals who met the inclusion criteria were consented and were assigned a unique identification number (UIN) to anonymize the data. Password-protected tab-delimited electronic databases were used to collate all data sets, and the UIN was used to link related data. The following inclusion criteria for participants was used: healthy individuals, and those with pre-existing comorbidities and medical conditions, aged ≥18 years planning travel to countries outside Europe, North America, and Australia for a duration of 2 weeks to 3 months.

The following exclusion criteria was also applied:

1. Travel outside of Europe, North America, and Australia 3 months prior to recruitment or planned travel within 6 months after return.
2. Antimicrobial use at time of recruitment or within the previous 2 weeks.

Participants provided a stool specimen and completed a questionnaire pre-travel, within 2 weeks post-travel, and 5–7 months after travel (follow-up).

A total of 107 participants were recruited to ensure sufficient sample donation and questionnaire completion at all three time points were obtained for 50 participants. Stools of the first 50 participants for which complete sample sets were available were prepared for sequencing. The median age for these 50 individuals was 41.5 years (range 22 to 78) and 34 (68%) were females. Fifteen individuals (30%) suffered from chronic conditions, including extraintestinal chronic health complaints such as asthma, hypertension, psoriasis, cancer, diabetes, HIV, osteoporosis, and gastrointestinal conditions, including gastric ulcers, irritable bowel syndrome, and inflammatory bowel disease. Three (6%) were pregnant during their first visit. Nineteen (38%) individuals had traveled to Sub-Saharan Africa, 10 (20%) to Southeast Asia, 15 (30%) to Central/South America or the Caribbean, six (12%) to the Indian subcontinent. Median length of journey was 18.5 days (range 14 to 78).

Twenty-three individuals (46%) experienced diarrheal episodes during their trip. Symptoms lasted less than a week in most individuals affected by TD (*n* = 21), one participant reported a duration of 10 days and another a duration of 42 days. Four participants who suffered from TD additionally experienced fever, a further four nausea and vomiting, one muscle cramps, and one flu-like symptoms. Eleven (22%) reported antibiotic use during or immediately after travel. Of these, nine (82%) used ciprofloxacin and two (18%) used azithromycin. Of the 11 participants using antimicrobials, eight took them for a single day, one completed a 2-day, one a 3-day, and one a 6-day course. All but one participant who used antimicrobials suffered from TD. Eight (16%) individuals took antibiotics between post-travel and follow-up visit. Despite the exclusion criteria, 10 (20%) subjects re-traveled outside Europe, North America, and Australia before their follow-up visit (Table S1S). To prevent overall sample numbers from becoming too low, they were not excluded from the study, despite planned repeat travel initially being an exclusion criterion.

### Ethical approval.

Regulatory and ethical approvals were obtained from the London Research Ethics Committee (Ref 15/0717; ID 189518). Informed written consent was provided by all study participants.

### Sample preparation.

Pre-paid return sample transport boxes for mailing of category B biological substances were given to all participants, including stool sample collection instructions. Participants were advised to return the stool sample as soon as possible (within 24 h), either directly to the clinic that day or alternatively at a designated collection point. Fecal specimens were stored frozen at –80°C, batched, and sent to the Gastrointestinal Bacteria Reference Unit, Public Health England (PHE, now UKHSA), Colindale, for DNA extraction.

### DNA extraction.

Total genomic DNA was extracted using a mixture of mechanical and enzymatic lysis. Dietary residue was removed by incubation with 2% 2-mercaptoethanol and sterile filtration. Human DNA depletion was carried out by selective lysis of human cells and DNase treatment using the MoLysis kit (Molzym, Bremen, Germany). Enzymatic lysis was carried out using the MasterPure purification kit (Epicentre, Madison, Wisconsin) with additional incubation with lysozyme (10 mg/mL), mutanolysin (20 U/μL), and lysostaphin (8 μg/μL). Mechanical lysis was carried out by bead beating with a FastPrep instrument (MP Biomedicals, Santa Ana, California) followed by a protein precipitation step and DNA precipitation with isopropanol. Extracted DNA was stored at –20°C until sequencing.

### Metagenomic sequencing.

Library preparation and sequencing were completed by MicrobesNG (University of Birmingham, Birmingham). Metagenomic DNA libraries were generated using the Nextera XT kit (Illumina, San Diego, California). Paired-end 125 bp sequencing was performed on a HiSeq instrument (Illumina, San Diego, California) in high-output mode.

Post-host DNA removal, per-sample read counts ranged from 97,538 to 112.20 million with a median of 37.28 million reads per sample. One pre- and one post-travel sample were excluded due to low yield counts. The samples were from two different participants, both of which re-traveled and one of which experienced TD and used antibiotics. All three samples from these participants were excluded from the following analyses, reducing the total number of complete data sets to 48.

### Bioinformatic processing.

Trimming of paired-end reads and removal of adapter contamination was performed with Trimmomatic, version 0.36 (36) with the following parameters: ILLUMINACLIP 2:20:10:3:TRUE, LEADING 3, TRAILING 3, SLIDINGWINDOW 4:15, MINLEN 36. Contaminant host DNA was identified by alignment of trimmed reads to the GRCh38.p13 human genome assembly (GCA_000001405.28) with the Burrows-Wheeler-Aligner (BWA) (37) and removed with Samtools (38). Remaining reads were aligned to the ResFinder database (version 2021-09-23) (22) using Bowtie2 (39). To control for ambiguous mapping the ResFinder database was clustered at 90% sequence identity using CD-HIT (40) and read counts aggregated to this stratification as performed by Munk et al. (41). Raw counts were converted to FPKM fragments thereby adjusting for read length and per-sample total bacterial sequence abundance. For virulence profiling of E. coli reads were aligned to the E. coli virulence finder database also clustered at 90% sequence identity using CD-HIT and read counts aggregated to this stratification, raw counts were aggregated to FPKM as above. Microbial taxanomic profiling was performed using MetaPhlAn 3.0 (42) with read counts output per clade and aggregated to the species level. StrainPhlAn 3.0 (42) was used for E. coli strain profiling from the metagenomic reads against a reference set of public genomes spanning the E. coli phylotypes and pathotypes (43). Pathotypes were defined as EPEC based on the presence of *eae*/*bfpA*, EAEC based on the presence of *aaiC/aggR*, ETEC based on the presence of *ltcA/sta1/stb*, EIEC based on the presence of *ipaH*, and STEC based on the presence of *stx*_1_/2.

### Statistical analysis.

Differences in the number of detected ARDs and abundance at different time points were assessed using the Wilcoxon signed-rank implemented in SciPy v1.7.1 (https://scipy.org/). Differences in the abundance between participants based on the use of an antimalarial prophylactic, the use of antibiotics during travel, and whether the participant had TD were assessed using the Mann-Whitney U test also implemented in SciPy v1.7.1. Changes in proportion of antimicrobial resistance classes and gene clusters in the pre-, post-, and follow-up samples was assessed using the McNemar's test implemented in the statsmodels v0.14.0dev0 python package. A *P*-value ≤0.05 was considered statistically significant for all tests.

### Alpha and beta diversity and ordination.

Alpha diversity measures of both resistome and microbiome was calculated using the Phyloseq (44) package in R and significance estimated using the Wilcoxon signed-rank test. Beta diversity was assessed using the adonis test implemented in Vegan (45). Ordination was performed using NDMS using the metaMDS function implemented in Vegan (45).

### Differential abundance testing.

Differential abundance testing of microbial taxa or antibiotic resistance elements was performed using ANCOM-BC (46). For the comparison between pre- and post-travel samples, structural zeros were imputed under the assumption that discrete sets of antimicrobial resistant genes circulate in high- and low-risk countries (47). For all comparisons, the library cut-off was set to 0 and the zero cut-off set to 0.97 and *P*-values adjusted using the Holm methodology. Adjusted *P*-values ≤ 0.05 were deemed significant.

### Data availability.

All short-read sequencing data were submitted to the NCBI Sequence Read Archive BioProject PRJNA872328 and individual accessions are listed in Table S1.

The authors confirm all supporting data, code and protocols have been provided within the article or through supplementary data files.
