# Supplementary material for: Prevalence and Persistence of Antibiotic Resistance Determinants in the Gut of Travelers Returning to the United Kingdom is Associated with Colonization by Pathogenic Escherichia coli
Source: Microbiol Spectr. 2023 May 31;11(4):e05185-22. doi: 10.1128/spectrum.05185-22 (PMC10433802; doi:10.1128/spectrum.05185-22)
Supplement: Supplemental file 1 — Fig S1. Download spectrum.05185-22-s0001.pdf, PDF file, 0.3 MB [file spectrum.05185-22-s0001.pdf]

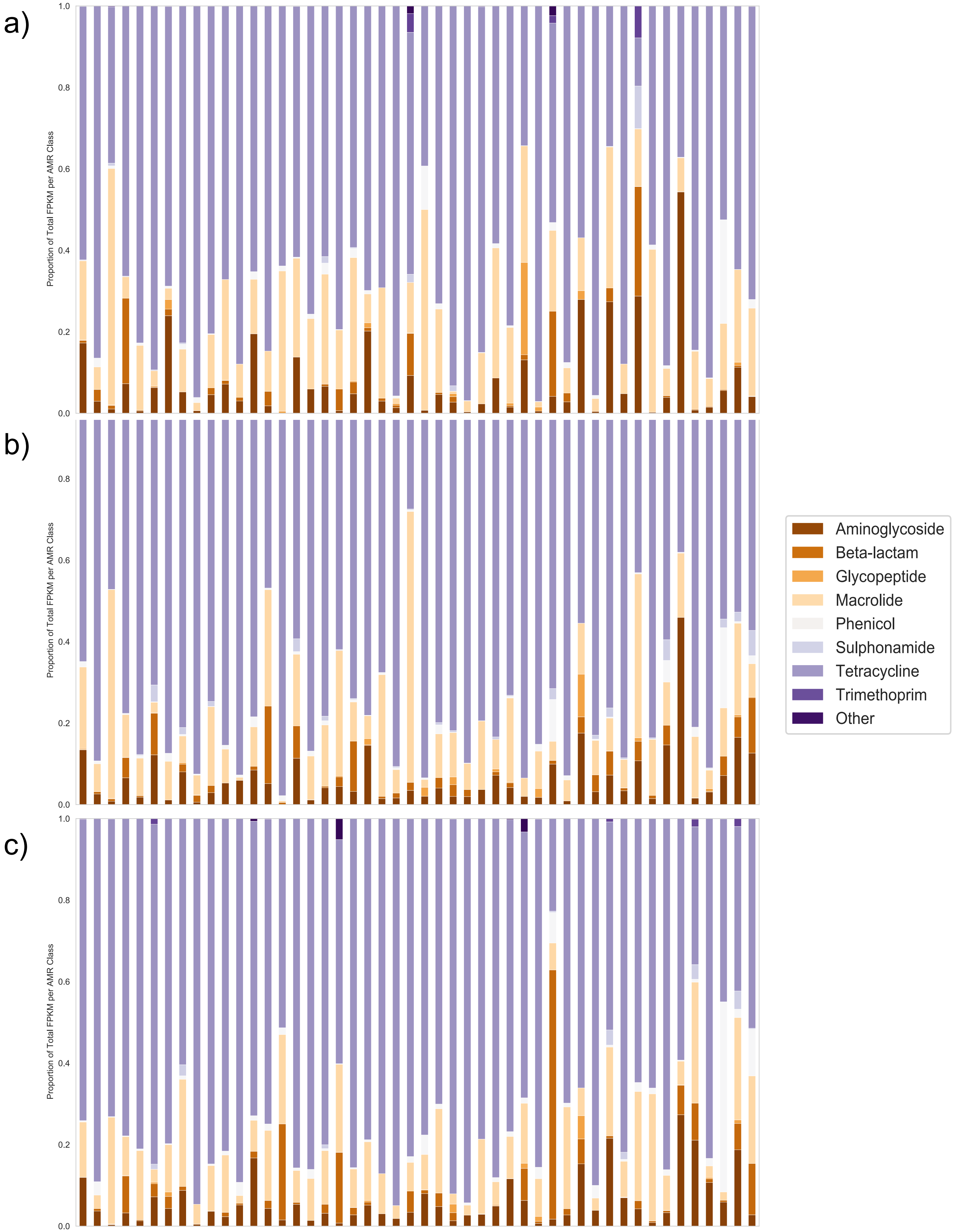

Supplementary Figure 1. Proportion of resistome per antimicrobial class, per sample, per specimen type (a=Pre Travel, b= Post Travel, c= Follow up)
