# Supplementary material for: Prevalence and Persistence of Antibiotic Resistance Determinants in the Gut of Travelers Returning to the United Kingdom is Associated with Colonization by Pathogenic Escherichia coli
Source: Microbiol Spectr. 2023 May 31;11(4):e05185-22. doi: 10.1128/spectrum.05185-22 (PMC10433802; doi:10.1128/spectrum.05185-22)
Supplement: Supplemental file 3 — Fig S3. Download spectrum.05185-22-s0002.pdf, PDF file, 0.08 MB [file spectrum.05185-22-s0002.pdf]

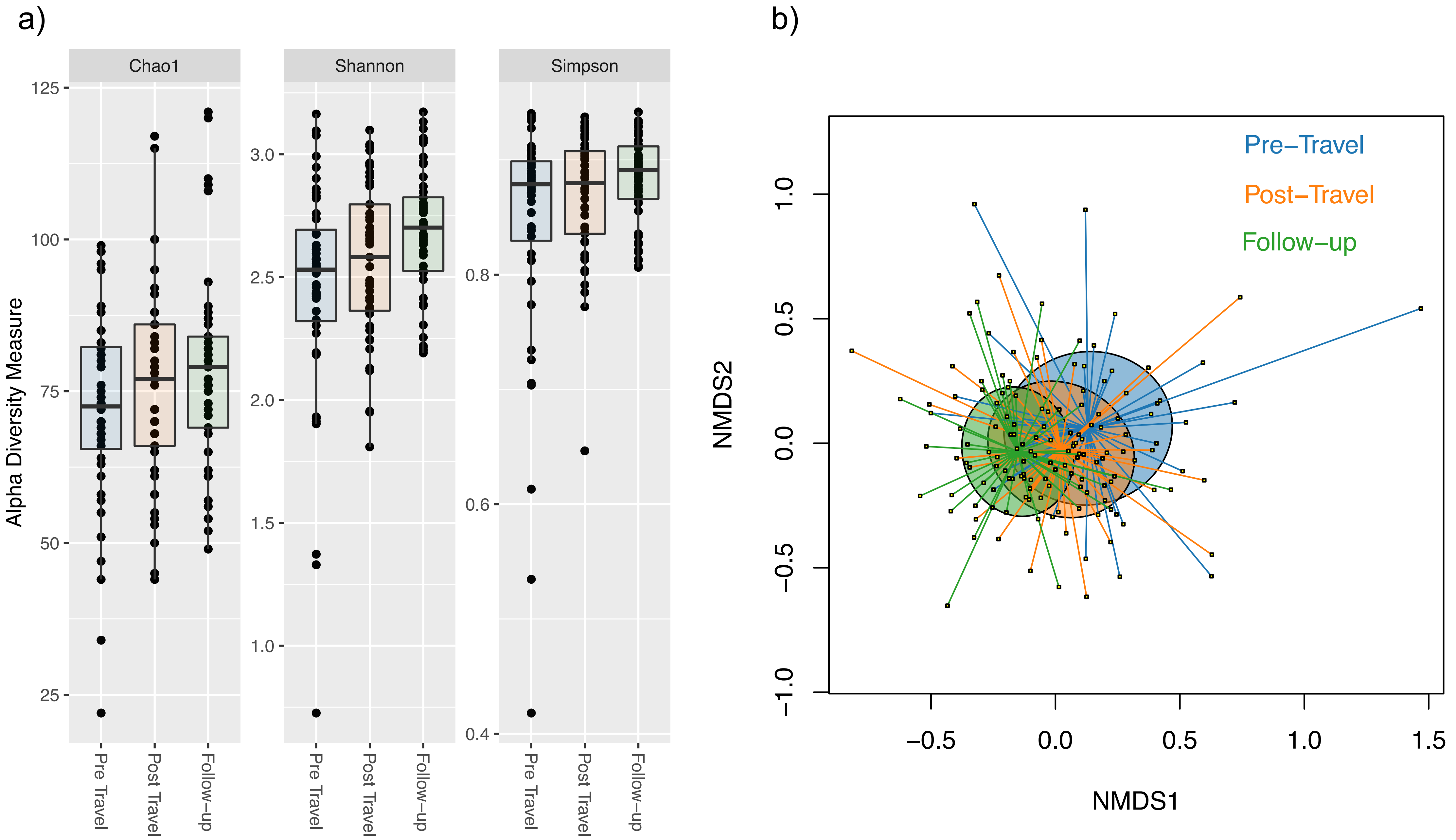

Supplementary Figure 3. A) Alpha diversity of microbiome per sample type based on Chao1, Shannon and Simpson's metrics. B) NDMS ordination of microbiome composition based on specimen type.
