# Supplementary material for: Prevalence and Persistence of Antibiotic Resistance Determinants in the Gut of Travelers Returning to the United Kingdom is Associated with Colonization by Pathogenic Escherichia coli
Source: Microbiol Spectr. 2023 May 31;11(4):e05185-22. doi: 10.1128/spectrum.05185-22 (PMC10433802; doi:10.1128/spectrum.05185-22)
Supplement: Supplemental file 4 — Fig S4. Download spectrum.05185-22-s0003.pdf, PDF file, 0.3 MB [file spectrum.05185-22-s0003.pdf]

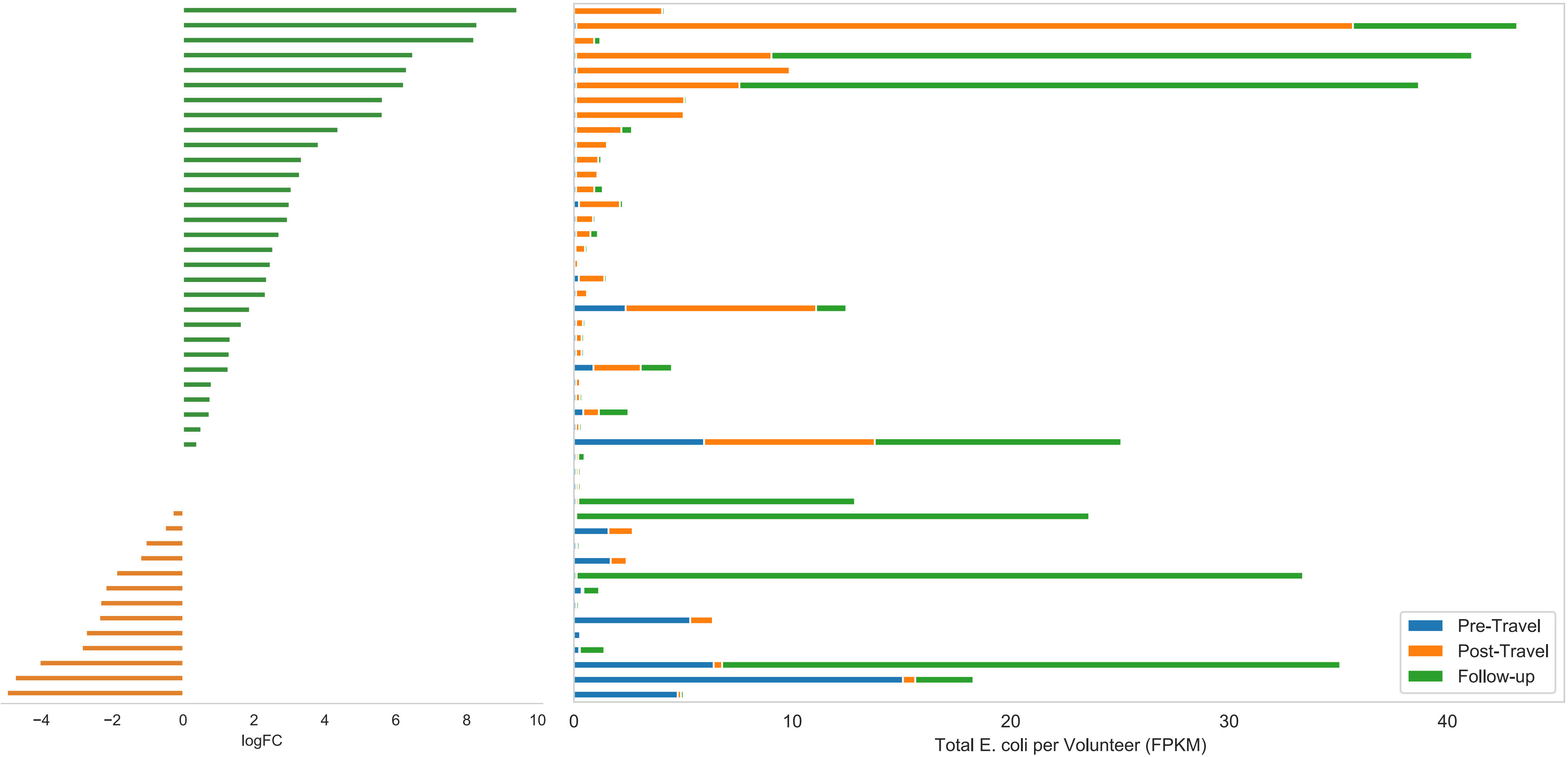

Supplementary Figure 4. Difference in *E. coli* abundance in the post-travel specimen compared to the pre-travel specimen for each participant expressed as $\log_2$ fold change and total *E. coli* abundance expressed by Fragments Per Kilobase reference per Million bacterial fragments (FPKM) within the pre-Travel, post-Travel and follow-up samples.
