# Supplementary material for: Prevalence and Persistence of Antibiotic Resistance Determinants in the Gut of Travelers Returning to the United Kingdom is Associated with Colonization by Pathogenic Escherichia coli
Source: Microbiol Spectr. 2023 May 31;11(4):e05185-22. doi: 10.1128/spectrum.05185-22 (PMC10433802; doi:10.1128/spectrum.05185-22)
Supplement: Supplemental file 2 — Fig S2. Download spectrum.05185-22-s0004.pdf, PDF file, 0.05 MB [file spectrum.05185-22-s0004.pdf]

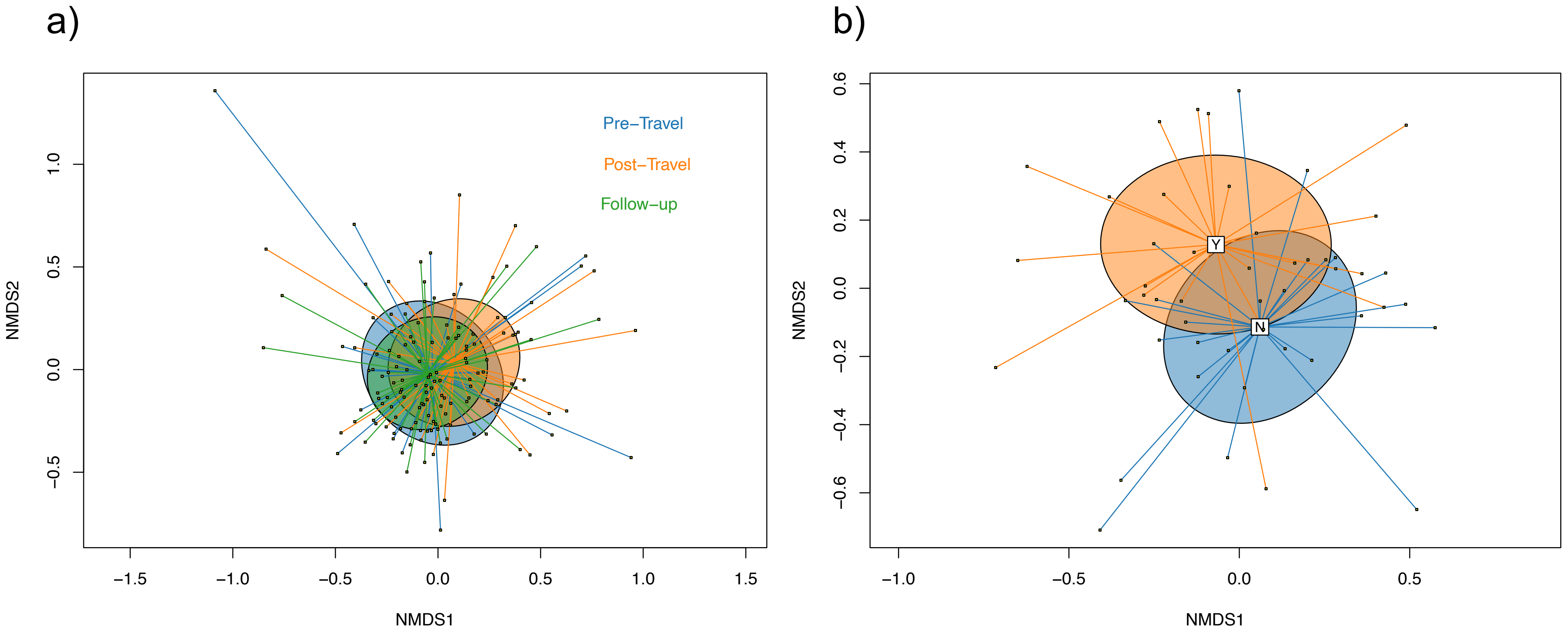

Supplementary Figure 2. NDMS plots showing A) ordination of resistome composition based on specimen type and B) ordination of post-travel resistome composition by occurrence of travellers' diarrhoea (Orange TD observed, Blue TD not observed).
